# The EU Nature Restoration Regulation offers new opportunities for resilient forests and sustainable forestry

**DOI:** 10.1007/s13280-025-02309-3

**Published:** 2025-12-22

**Authors:** Johan Svensson, Bengt Gunnar Jonsson, Torbjörn Ebenhard

**Affiliations:** 1 Department of Wildlife, Fish and Environmental Studies, Faculty of Forest Sciences, Swedish University of Agricultural Sciences, 901 83 Umeå, Sweden; 2 Department of Natural Sciences, Design and Sustainable Development, Mid Sweden University, 851 70 Sundsvall, Sweden; 3 Department of Urban and Rural Development, Swedish Biodiversity Centre, Swedish University of Agricultural Sciences, Box 7016, 750 07 Uppsala, Sweden

**Keywords:** Active restoration, Climate change adaptation, Continuous cover forestry, Financing restoration, Multiple-use forestry, Private forest owners

## Abstract

This paper examines the EU Nature Restoration Regulation (NRR) as a novel framework aimed at promoting biodiversity, ecosystem services, sustainable forestry practices and resilient ecosystems. We highlight the Swedish forests’ pivotal role in realizing the NRR goals given the substantial forest area and forest value capital, as well as the urgent need for ecological restoration, diversified forest management, and climate change adaptation in forestry. We highlight key NRR provisions, particularly Articles 4 and 12 that set quantitative restoration targets for forest habitat types and general restoration practices in forests. We note political resistance manifested in conservative interpretations that risk to compromise conservation and sustainable forest management in the future. Finally, we consider strategic, tactical, and operational NRR implementation challenges, emphasizing the importance of a comprehensive national restoration plan that integrates scientific evidence on restoration needs and the financial opportunities to secure long-term ecological and economic benefits for forest owners.

## Introduction

### Implementing the EU Nature Restoration Regulation intentions

New opportunities for European forests and forestry arise with the intentions in the EU Nature Restoration Regulation (NRR; EC 2024). If inventively steered toward holistic perspectives and taking all values associated with forests, forest resources and forest landscapes into account, the NRR can assist the EU Member States’ forest-sectors to navigate toward functional, resilient forest ecosystems and sustainable, future-oriented forest governance and management. Sweden harbors a key share of European forests (12%, Forest Europe 2020) with substantial economic, ecological, and sociocultural capital and is thus well-suited to take a leading role in the Unions’ implementation of the NRR. Furthermore, as the ecological restoration needs are evident in the Swedish forest and forest landscapes following an era with extensive industrial rotation forestry (e.g., Chapron 2022; Swedish Forest Agency 2022; Svensson et al. 2023; EC 2025), progressive routes that embrace future sustainable forest and forestry opportunities arising with the NRR are urgently required.

Successful implementation of the NRR relies on its national uptake and implementation; the Member States have the implementation mandate. Given resistance toward the adoption of the NRR from some States, such as signaled from the Swedish government, we are concerned that its intentions are jeopardized. A minimum-level interpretation not based on scientific evidence may put Sweden’s reputation as an advanced forest nation at risk, negatively influence a creative uptake of the NRR among other Member States, and risk Swedish forest sector conflicts with the EU. Also importantly however, a reactive and resistant direction delays a necessary transformation of Swedish forestry to raise the capacity for climate change adaptation, biodiversity conservation, and viably balancing the rights of multiple forest and forest landscape stakeholders. With this background, the purpose of this paper is to provide perspectives on how the NRR can be implemented in Sweden, specifically focusing on areas in need for restoration and on forest management and planning options. Here, we take a holistic perspective of the Swedish forest landscape, including the potential future role of non-industrial private forest owners and opportunities for funding restoration.

### The current state of Swedish forests and forestry

Forest land in Sweden amounts to 69% of the land area and has been managed with a focus on wood biomass production for centuries (Swedish Forest Agency 2020). A total of 19.6 million ha is considered as “timber production land”, defined as productive forest sites (a mean annual growth of merchantable wood per hectare over a rotation period of 1 m^3^ or more) that are logistically available, not under other land use, or protected (SLU 2024). Current forest management on timber production land implies clear-cutting of single-age monoculture stands in rotation intervals of 60–90 years, with Scots pine (*Pinus sylvestris*) and Norway spruce (*Picea abies*) currently making up about 80% of the standing biomass (Ibid.). With minor exceptions, forestry is carried out in the same way everywhere, at all scales, levels, and steps from seed to plank. Exceptions include, for example, higher nature conservation ambition on 111 000 ha within Sveaskog State Forest Company Ecoparks (Bergman and Gustafsson 2020) and an estimated 720 000 ha (3% of the productive forest land) with non-clear-cut forestry, primarily on forest land owned by non-industrial private persons (Swedish Forest Agency 2021, 2023b).

This simplified and systematic forestry model has led to successful expansion of timber production land and increase in wood biomass production levels (Swedish Forest Agency 2020; Ståhl et al. 2024), but also to a trivialization of forests and forest landscapes, loss of species (1400 threatened mainly caused by the clear-cutting practice; SLU Artdatabanken 2020) and biodiversity, increased vulnerability and reduced sustainability (e.g., Angelstam 2022). Seen over the past decades, the harvesting level has increased relative to the growth, the forest's carbon sink decreased, the tree-growth slowed down, and an increasing share of stands clear-cut at an age younger than the regulated youngest age (Swedish Forest Agency 2022, 2023a, 2024; Fridman and Nilsson 2025). Forests experience impact of droughts, storms, fungal and insect infestations as well as hard-to-control fires in even-aged coniferous forest monocultures.

Open and semi-open land with their specific biological and cultural historical values are disappearing (Eriksson 2018). Indigenous rights associated with reindeer husbandry and other traditional cultures and land uses in forests and forest landscapes are only marginally considered (Beland Lindahl et al. 2017; Neumann et al. 2022; Saito et al. 2023; Kløcker Larsen et al. 2024). Only fragmented remnants of natural forests with high conservation values and intact forest landscapes remain (Svensson et al. 2020), with forest harvesting continuing at high rate also in these (e.g., Jonsson et al. 2019).

Despite the equal portal objectives on production and environment, the Forestry Act has a base in production forestry and on sustained wood-biomass yield (Swedish Forest Agency 2020). Environmental considerations are limited, and in practice it is the more far-reaching requirements of the forest certification standards that apply (Lehtonen et al. 2021). Forestry planning is binary at stand management-units with a focus either on production goals or, to a minor extent, on nature conservation goals (Swedish Forest Agency 2020). Other forest and forest landscape values and interests are not considered in planning following the standardized forest management goal classification scheme. Non-clear-cut forestry, as a complementary forest management model promoted by the Swedish Forest Agency (2021, 2023b), is still focusing on maintained wood biomass production goal on stand level, with harvesting regulations following average tree height and volume functions for single-cohort coniferous tree species.

The uniformity in management planning fails to acknowledge practices and value chains that reflect the multi-functional capacity of forests, intrinsically relevant and important throughout history (Zhang et al. 2022). Diversification is lacking (Felton et al. 2020). The capacity and varying premises among non-industrial private forest owners, owning about 50% of the forest land and supplying some 60% of the material to the forest industry (Swedish Forest Agency 2020), are not captured to diversify provisioning of forest values, services, and goods. Instead, support and advice from forest owner associations with their own industry or from forest company timber buyers manifest the simplified and systematic production forestry model and limit market influence and diversification (Blattert et al. 2023). Neutral and independent advice only occurs to a limited extent (Carlsson et al. 2017; Lidestav and Westin 2023). The forest owner association´s role as representing the owners has, accordingly, been questioned (Swedish Forest Agency 2024).

Forest management planning on a larger geographical scale is realized only in special cases, such as in the Sveaskog Ecoparks (Bergman and Gustafsson 2021). Despite initiatives at county and national levels for a long time, the work on green infrastructure has not found its forms and currently lacks governmental support and funding (Angelstam et al. 2020). This limits ways forward to creating networks of forest habitats that ensure functional forest ecosystem connectivity.

Below the Scandinavian Mountains Green Belt (Svensson et al. 2020), the formal and voluntary protection of forest is not sufficient (9.2%; Statistics Sweden 2024) and thus falls significantly short of the targets set at EU level of 30% whereof 10% strictly protected (EC 2020). Strict protection should include all primary forest and old-growth forest, with appropriate management, clear goals, measures and monitoring. Consequently, Sweden does not meet either national, EU, or international environmental objectives (Swedish Forest Agency 2022; Angelstam et al. 2020). Criteria and measures of sustainability with regard to biodiversity, as adopted in the Kunming-Montreal Global Biodiversity Framework (CBD 2022), are not in place.

Hence, the need for restoration, diversification, and multiple use is evident in the Swedish forests and forest landscapes, as is the need to strengthen the mandate of private forest owners, a landscape perspective in forest management planning, and climate change adaptation. The current and historical forest land use in Sweden, as well as into a future with changing climate, markets and demands, calls for restoration of forests and forest landscapes at all scales and levels in line with or exceeding the NRR intentions.

## Core aspects of the Nature Restoration Regulation

### The regulation concerning forests

The NRR was adopted in June 2024 as part of the EU strategy for biodiversity (EC 2020). Articles 4 and 12 contain the most critical elements for forests, forestry and forest conservation. Implementing Articles 4 relies on an estimate of favorable reference areas for EU Habitats Directive Annex 1 habitat types (EC 2024), which according to the NRR should be based on best available scientific knowledge. The NRR defines the favorable reference area as the minimum area necessary at the national level to ensure the long-term viability of the habitat type and its typical species, and it is used in the NRR to set a lower threshold for restoration needs. The current favorable reference area estimates for Sweden were presented by the Swedish Environmental Protection Agency in 2024, following an organized process with input from scientific experts (Swedish EPA 2024).

**Fig. 1:**
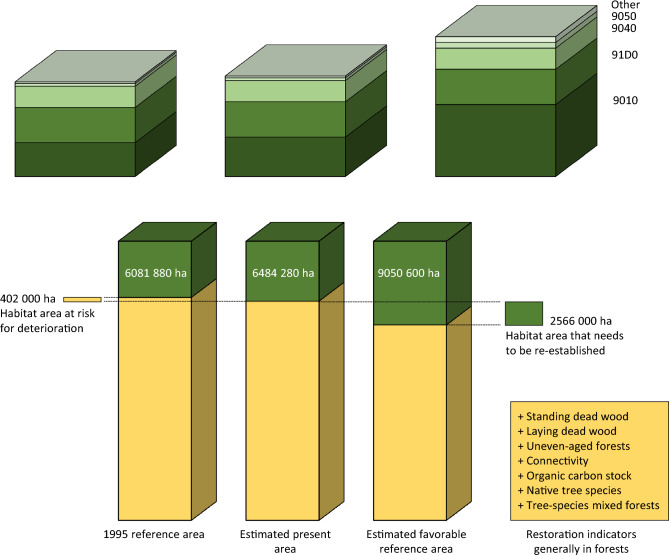
A comparison between reference areas based on the habitat areas existing in 1995, estimated present and estimated favorable reference areas for habitat types and general restoration needs in forests according to NRR Articles 4 and 12. Compared with the estimate of the present area of EU Habitats Directive Annex 1 habitat types (green, middle bar), the 1995 reference area (green, left bar) implies that 402 000 ha are surplus to conservation needs. Contrarily, the scientifically estimated reference area (green, right bar) implies that re-establishment of another 2566 000 ha (8.5% of forest area) is needed. The yellow area of the bars show estimated forest area without habitat-type qualities, but with general restoration needs as expressed by the NRR Article 12 indicators, as listed to the right. The mandatory indicator on common forest birds index is not included. The total area illustrated in the bottom panel bars equals total forest and woodland area (i.e., 30 178 000 ha; SLU 2024). The top panel bars show a division into area of the four most abundant habitat types with the remaining 11 types combined (other). The 1995 reference area for Western taiga (9010) equals 46% of the scientifically estimated favorable reference area for this habitat type and implies that 393 000 ha of its estimated present area is considered surplus. Data: Swedish Environmental Protection Agency (2024). EU Habitats Directive Annex 1 habitat types with 1995 reference areas, estimated present, and estimated favorable reference areas, in order of decreasing area, in ha: 9010 Western taiga; 2150 300; 2543 100; 4645 400. 91D0 Bog woodland; 2238 700; 2238 700; 2238 700. 9040 Nordic subalpine/subarctic forests with *Betula pubescens ssp czerepanovii*; 1392 100; 1392 100; 1392 100. 9050 Fennoscandian herb-rich forests with *Picea abies*; 200 000; 200 000; 401 500. 9080 Fennoscandian deciduous swamp woods; 28 600; 28 600; 108 500. 9030 Natural forests of primary succession stages of landupheaval coast; 17 000; 17 000; 17 000. 9160 Sub-Atlantic and medio-European oak or oak-hornbeam forests of the *Carpinion betuli*; 8300; 14 500; 80 000. 9020 Fennoscandian hemiboreal natural old broad-leaved deciduous forests (*Quercus*, *Tilia*, *Acer*, *Fraxinus* or *Ulmus*) rich in epiphytes; 11 800; 13 100; 16 500. 9110 *Luzulu*-*Fagetum* beech forest; 9700; 10 500; 37 000. 9190 Old acidophilous oak wood with *Quercus robur* on sandy plains; 5200; 6400; 59 000. 9060 Coniferous forests on, or connected to, glaciofulvial eskers; 6000; 6000; 20 000. 91E0 Alluvial forests with *Alnus glutinosa* and *Fraxinus excelsior* (*Alno-Padion*, *Alnion incanae*, *Salicion albae*); 6000; 6000; 17 000. 9130 *Asperulo-Fagetum* beech forest; 5200; 5300; 12 100. 9180 *Tilio-Acerion* forests of slopes, screes and ravines; 2140; 2140; 5000. 91F0 Riparian mixed forests of *Quercus robur*, *Ulmus laevis* and *Ulmus minor*, *Fraxinus excelsior* or *Fraxinus angustifolia*, along the great rivers (*Ulmenion minoris*); 840; 840; 900

Article 4 concerns habitat types in the EU Habitats Directive, whereof 15 forest habitat types occur in Sweden. The current estimated areas of these habitat types, except two (Bog woodland; Nordic subalpine/subarctic forests with *Betula pubescens ssp czerepanovii*) are below their favorable reference areas (Swedish EPA 2024). Hence, for the remaining 13 types there is a need to re-establish new forest areas to fulfill the requirements. The total present estimated area of the 15 habitat types is close to 6500 000 ha across the alpine, boreal, and continental biogeographical regions, with estimated re-establishment needs corresponding to 2600 000 ha (Fig. 1). This will generate an area equal to 9050 600 ha. In addition to re-establishment, restoration is needed to improve the status of existing habitat areas with unfavorable conditions.

Article 4 sets quantitative targets for restoration of the existing habitat area that are in unfavorable condition, targeting a minimum of 90% in good condition by 2050. Article 4 also calls for re-establishment of habitat area up to 100% of the favorable reference area by 2050, with milestones in 2030 (30%) and 2040 (60%). For habitat types that already cover more than 3% of the total national land area, slightly lower percentages may be used as targets. If it is considered impossible to re-establish up to the reference area, 90% of the area can be accepted.

In habitat areas with existing good conditions, in areas that are restored and in areas planned for re-establishment of the habitat type, Article 4 calls for measures (not specified) that aim to prevent significant habitat deterioration. Such measures may be applied on a biogeographical region level instead of at the level of delineated forest patches. The NRR does not clarify, however, what should be regarded as significant deterioration at the regional level. There are also general clauses that allow Member States to deviate from the non-deterioration requirement, with regard to natural disasters, habitat impact caused by climate change, action or inaction by another Member State, or if restoration is conflicting with critical societal functions such as renewable energy and national defense installations (Articles 6 and 7).

Whereas article 4 concerns the EU Habitats Directive and the conservation of threatened habitat types, Article 12 is directed toward restoration in forest ecosystems in general, including timber production land. A general paragraph calls for restoration measures necessary to enhance biodiversity, without further elaborations, implying that forest management generally should achieve ecological sustainability. While the NRR does not set specific sustainability criteria, Article 12 lists seven indicators that should show positive trends by regularly reported monitoring based on the restoration measures taken. One mandatory additional indicator is the common forest birds index, which should show a positive trend from 2024 until satisfactory levels are reached. Member States are also expected to orient the national restoration plans toward a minimum of six of the indicators (Fig. 1) and monitor and report a positive trend in those. Again, to achieve satisfactory levels, as defined by each Member State based on scientifically established knowledge.

A third article of interest, Article 13, calls for Member States to contribute to the union-wide goal to plant an additional three billion trees. Planting shall take into account factors such as age structure, native tree species, ecological contexts, local habitat conditions, increased resilience to climate change, and improved ecological connectivity. It is specifically stated that accurate scientific knowledge should be applied to establish not only reference areas for habitat types, but also that restoration should involve afforestation of land not currently forest land. Furthermore, restoration should be directed to lands that are best-suited to respond to the restoration measure, and to locations where ecological connectivity would be improved, also taking synergies with agriculture land and climate change into account (Article 14).

Member State governments will submit a draft national restoration plan to the EU Commission for evaluation (Article 17). The Commission may request Member States to justify the level of ambition. Non-compliance may result in a risk for serious criticism and forcing legal actions.

### Implementation of the regulation in Swedish forests and forest landscapes

The Swedish government voted against the NRR in the EU Council. The main line of contention was Article 4, especially regarding the habitat types Western taiga and Fennoscandian herb-rich forests with *Picea abies*. Estimated present areas are 2540 000 ha and 200 000 ha, whereas favorable reference areas are 4600 000 ha and 402 000 ha, respectively (Swedish EPA 2024); hence, a substantial re-establishment is required (Fig. 1). Both habitat types are of specific interest for forestry due to their productive capacity and abundance, particularly in the boreal region which has a predominance of private forest company land ownership (Jonsson et al. 2019).

The NRR has entered into force as a law in Sweden as in all EU Member States. The Swedish government has commissioned the Swedish Environmental Protection Agency and other government agencies to draft a national restoration plan (Swedish Government 2024a), as stated in NRR Articles 14 and 15. The government instructions include to apply a minimum requirement level and make use of the flexibility allowed in the NRR articles to its full extent, to ensure as low costs and negative impact as possible for Swedish forest sector enterprises (Ibid.).

The Swedish government has also instructed the Swedish EPA (Swedish Government 2024b) to, when the agency submits the 2025 Article 17 report according to the EU Habitats Directive, to apply as a favorable reference areas for forest habitat types the areas estimated as present in 1995, i.e., at the time of Sweden joining the EU. This disregards the definition of favorable reference area in the NRR, as a scientifically based area needed to ensure the long-term viability of the habitat types and their typical species. Applying the 1995 areas as reference areas in the Swedish national restoration plan would mean that Sweden would not need to re-establish any area of the 15 forest habitat types. Instead, a total of over 400 000 ha of existing habitat area, almost exclusively consisting of Western taiga, would be regarded as surplus to the reference area requirement and hence risk deterioration.

Furthermore, the government has commissioned the Swedish EPA to revise the national interpretation of the EU habitat-type manual, i.e., to re-define the criteria of each of the listed forest habitat types. The consequence of such revised definitions, regarding the EU Habitats Directive, may be that an evaluation of conservation status as unfavorable converts into favorable, simply because the criteria that determine the quality of each habitat type are modified or removed. Such a potential habitat devaluation should be considered in the view of a very large share of the forest habitat types currently being in unfavorable condition (94%; EC 2025). Consequently, this may lead to further loss of habitat area and reduced areas with apparent restoration needs, and thus to an elevated risk to not adhere to the Article 4 non-deterioration requirements. Since the NRR focuses both on habitats (Article 4) and on forests in general (Article 12), any reduction with respect to Article 4 favorable habitat reference area will further stress the need for far-reaching Article 12 restoration in forests in general, including on timber production land. Since the timber production land is predominantly represented by management-modified forests, this implies that positive ecological response to restoration may be delayed due to extensive restoration needs and a prolonged ecological recovery time, with subsequent depreciation effects on regular NRR monitoring, reporting and verification. Consequently, resources would instantly have to be allocated to developing knowledge to conduct and validate a scientifically sound approach to such massive restoration effort, instead of focusing on restoring existing more natural or semi-natural forests with a shorter ecological delivery time. Defining such scientifically sound approaches is an arduous task, given that research and knowledge on positive biodiversity effects of ecological restoration and alternative forest management systems are still fragmented or lacking (Hertog et al. 2022; Larsson Ekström et al. 2024; Ståhl et al. 2024).

If the Swedish government maintains its current views on habitat reference area, requirements regarding forest habitat types covered in NRR Article 4 may actually be eliminated. If this strive to limit the impact and consequences in the Swedish forestry sector results in setting arbitrary low levels of the Article 12 indicators as satisfactory, the intentions of the NRR may be lost. Some of these indicators do show increasing trends in Sweden, due to measures imposed by forest certification schemes. For example, dead wood is slowly increasing, however from a very low level to far from representative in natural conditions (Siitonen 2001). Given such a low NRR implementation strategy, the inventive capacity of the NRR could similarly be ignored by any Member State that takes lightly their commitments to biodiversity conservation, sustainable use in context of multiple rights associated with forests and forest landscapes, and adaptation of forestry to climate change.

## Prospects for a future-oriented Swedish sustainable forest model

### Strategic, tactical, and operational premises

The NRR incentivizes a new direction for sustainable forestry following a global and EU movement toward greater environmental concern and regulation. Restoration in a wide sense, with its historical development in Europe from disaster reduction up to the 1940s, increased forestland area and wood production, and multifunctionality beyond the 1990s (Erdozain et al. 2025), is now manifested as a component in forest governance and management models. However, even with a fact-based focus on sustainable forest management within a conservation and restoration frame, supported by research and monitoring, it is challenging to achieve the NRR goals and intentions. The Regulation targets large forest areas, forest owners are affected, forest governance and management systems need to be adjusted, and the prevalent forestry model and forest-product value chains must be diversified. A constructive ambition and spirit is needed to achieve the intentions of the NRR. Swedish forests and forestry, regardless, must adapt to forest and forest landscape stakeholder rights, new market demands and to a changing climate (Swedish Forest Agency 2023a).

The NRR has been set up to comply with international agreements, such as the Convention on Biological Diversity, the EU forest and biodiversity strategies, the EU Habitats Directive, and is in line with the UN Declaration of the ecosystem restoration decade (UNEP/FAO 2020). Since the progress in the implementation of these agreements is too slow (Secretariat of the Convention on Biological Diversity 2020; European Environment Agency 2025), this highlights the importance of setting a high level of ambition for the NRR favorable reference areas and indicators. Ignoring scientific evidence, setting artificial reference areas and habitat criteria does not change the degraded state of the natural values in forests per se. With a direct reference to the low favorable reference area politically decided, it must be realized that it is economically and ecologically more effective to meet the requirements of the NRR by keeping and restoring existing old growth, primary and Natura 2000 habitat forests, than to restore transformed forests that are removed from the timber production share of the Swedish forest landscape.

Given the purpose of the NRR, restoration should be steered to where it is ecologically most efficient (cf. Wang et al. 2025). Protection of forests that have high conservation value should be strengthened, and authorities, forest owners, and forestry practitioners should be provided with support in spatially identifying forest patches and landscapes where restoration will generate expected output.

In the Swedish forestry regulation system, only harvesting in the form of clear-cutting needs to be notified to the Swedish Forest Agency (Swedish Forest Agency 2023c). Clear-cutting can be prohibited within 6 weeks, yet not formally approved. Given that comprehensive field-validated knowledge of location and status of high conservation value forests in Sweden is lacking (Bubnicki et al. 2024), clear-cutting may thus not be prohibited despite existence of such values. If forest attributes  are in line with criteria for listed habitat types, the Habitats Directive, as reflected in the Swedish Environmental Code (1998) and the Forestry Act (Swedish Forest Agency 2023c), still applies. Thereby, conflicts hindering or stopping planned or ongoing harvesting operations may appear, that directly impact forestry planning and timber flow processes as well as put forest owners, forest machine operators and other directly involved persons in difficult situations. Successful implementing of the NRR ultimately calls for policy, governance and management strategies that strengthen the planning capacity to identify forest with habitat qualities and restoration potential, to avoid such direct conflict situations.

Activities associated with the public right of access (Saito et al. 2023) and hunting (Neumann et al. 2022), are examples of traditional values that cover close to 100% of the forests in Sweden and need to be secured to realize sustainability. Future forest governance and management requires a planning system that simultaneously addresses multiple values, including restoration to improve the conditions for the prioritized value or combination of values. Further, the planning system needs to be adaptive, dynamic and flexible over a rotation period, to adjust to climate change, favor native tree species, or meet new demands on forests. Multiple-use planning can include timber production balanced against other values.

Each year, about 400 million saplings are planted in Sweden as part of regeneration following clear-cutting (Swedish Forest Agency 2025). This cannot be accounted for as additional as intended in NRR Article 13. Afforesting the remaining fragments of open and semi-open grasslands and meadows would account but would harm essential biodiversity, cultural heritage and aesthetic values associated with such biotopes (Eriksson 2018). Hence, the Swedish ambitions need to be sensitive to such consequences in rural landscapes. The main option of planting additional trees in Sweden would instead be to focus on urban and peri-urban areas.

The Swedish Forest Agency is promoting closer-to-nature forestry as a more holistic approach to forest management (Swedish Forest Agency 2023b). Natural disturbance-based approaches can be integrated following, e.g., Berglund and Kuuluvainen (2021) while also reflecting other values and rights such as indigenous Sami reindeer husbandry in northern Sweden. Based on principles of continuous cover forestry (Mason et al. 1999), a way forward to maintain forest production while simultaneously promoting multiple-use, sustainability, landscape perspective and climate change adaptation, is to decrease the use of clear-cutting and increase the use of alternative forest management systems. Prolonged rotation periods will potentially increase the carbon storage capacity in forests (Schulte et al. 2022; Englund et al. 2025) compared with successivley shorter clear-cut rotation periods. Given that a large share of timber production land currently consists of even-aged monoculture coniferous stands, the transition to continuous cover forestry will take time (Hertog et al. 2022; Ståhl et al. 2024), particularly as forestry machines, operational planning and harvesting systems, timber buying and price lists routines, etc., are streamlined to the predominant forestry model practices. Intentionally acknowledging and planning for this transition represent a significant step to safeguard the uptake of the NRR in Sweden to the benefit of European forest and forestry capital.

Non-industrial private forest owners are central actors in forest restoration. In total 309 000 physical persons own 11 329 000 ha (productive forest land) in 232 000 management units with an average and median forest area of 34 ha and 11 ha, respectively (Swedish Forest Agency statistics 2025). Few forest owners (about 8%; Ludvig&Co 2023) have their main income from forestry; 32% of the forest owners hold up to 5 ha, 63% up to 20 ha and 93% up to 100 ha (Swedish FA 2025). At the same time, the ownership profile is changing into being characterized by older persons, fewer individual owners, an increasing share of remote resident owners, and an increasing share of capital investment owners (Ludvig&Co 2023). Is large-scale industrial rotation forestry the best way forward for all these owners? Or can we make the assumption that future ambassadors of conservation, alternative forestry and restoration can be found among these? The NRR emphasizes greater transparency and predictability, which ultimately must be manifested in advice and extension campaigns directed to forest owners, forestry managers and planners. There is a strong need for neutral advisors—brokers (Carlsson et al. 2017)—that engage in dialogue with forest owners, make sure that they have objective knowledge and insights, and support their views and interests in negotiating the tactical and operational forestry actions (Curtis et al. 2023).

### Financing restoration

Active restoration management in forestry is a necessity, for climate adaptation and for strengthening the existing natural values in protected forests where those values are supported by management intervention (e.g., Bernes et al. 2015). A positive trend in several of the Article 12 indicators can only be achieved with active measures. This often requires some wood extraction, with income opportunities for forest owners and timber flow to the industry. Alternative forest management methods often imply longer rotation periods and larger abundance of old and large trees (e.g., Mason et al. 1999), which with active management can increase harvesting share of valuable timber quality and decrease pulpwood, in addition to enhanced biodiversity and carbon storage values. Active restoration includes alternative forestry methods, but also restoration-adapted management in neglected pre-commercial and commercial thinning-stage forests. The Swedish Forest Agency (2016) reported that 2.5 million ha has been left unmanaged following previous clear-cutting. Such areas of plant-stage and young forests can be seen as potential low-cost restoration resources with, for example, thinnings oriented to favor deciduous dominated forests and forests with a mixture of tree species.

Yet, implementing the NRR requires financial resources. Is governmental funding primarily or solely presumed? If so, the already well-established system of nature conservation agreements, a formal protection instrument in Sweden since 1993, could be seen as a form of nature  credit (Svensson et al. 2025). These agreements are voluntary, based on dialogue, come with economic compensation, and with retained land ownership. They are, further, based on a documented presence of high conservation forest attributes, have a specified conservation goal, are valid for a certain time-period according to the Swedish Land Code (1970), and are known by forest owners and authorities. They can also be based on other values than nature conservation, such as recreational values. Active forestry measures can be agreed if in line with the conservation objectives, and the forest owner can sell harvested wood as part of the agreement. 

Nature conservation agreements on non-industrial private forest land across entire Sweden up to 2024 include a total of 5525 different small (1–221 ha; Svensson et al. 2025) forest patches together covering over 40 000 ha (Swedish Forest Agency statistics 2025). Parts of these are undergoing active restoration and other parts are set aside for natural development, i.e., passive restoration. Their contribution is important to the national forest conservation scheme, particularly in south Sweden where private owners dominate, and because of their nation-wide coverage across diverse and representative forest-habitat types. Nature conservation agreements are also applied on landscape scale such as for the nature conservation parts within the Swedish State Forest Company Sveaskog ecoparks. Across the Swedish land base, there are in total 37 Ecoparks ranging in size from 1200 to 22 400 ha, in total covering 250 000 ha land area (Bergman and Gustafsson 2021). The Swedish EPA and the regional county administrative boards have delineated larger landscape conservation core areas with a higher density of high conservation value forests than in the general forest landscapes (Wang et al. 2025). Nature conservation agreements could be used to create a network within these, in which “where to restore” planning approaches (Ibid.) could be applied to map how to improve connectivity and to identify what type of restoration would best support the local nature conservation and landscape values.

Another financing option would be to implement a fee on selling and buying wood. Such systems are already in place for other purposes. As an example, fees per 1 m^3^ (wood under bark) include for pulpwood 1.00 SEK to the Swedish Forest Institute, 0.28 SEK to forest industry communication campaigns, and 0.13 SEK to the national browsing survey by the Swedish Forest Agency (Holmen 2025). The same fees are applied for timber, with an additional 0.50 SEK to the Swedish Wood forest knowledge hub. Calculated based on harvested pulpwood and timber in Sweden in 2023 (31.4 million m^3^ pulpwood and 34.4 million m^3^ timber; Swedish Forest Agency statistics 2025), these fees equal 110 million SEK. A similar fee system could be considered to finance restoration. If a restoration fund could be founded based on such fees, it remains to be investigated how it can be best constructed to efficiently facilitate increased restoration in the right place and the right way (Svensson et al. 2025; Wang et al. 2025) to meet the NRR ambitions and NRP intentions.

Other expanding value chains than conventional forestry, such as biodiversity (Kim et al. 2025) and carbon credits (e.g., Probst et al. 2023), may provide marked-based funding opportunities for restoration more broadly, in addition to governmental funding, and support the development of a more diversified, competitive and dynamic market on forest resources and forest land. This would provide Swedish forest owners with options to choose among the different value chains or combinations of value chains that best meet their premises and interests, and the timber- and forest land- markets may better respond to open-market behavior.

## Conclusions

The Nature Restoration Regulation is part of a complex EU policy and legal framework directly connected with, for example, the Habitats Directive and the Biodiversity Strategy. The NRR applies as law in all Member States, in addition to the national legislation. Since NRR includes all ecosystems, there are also connections with other land covers and other legislation. The national restoration plan needs to reflect these contexts to allow policy- and decision makers, forest-product market actors, certification organizations, forest planners and managers, as well as forest owners to navigate toward sustainable forestry. Comprehensive implementation of the NRR offers a platform for avoiding silo-minded short cuts that would prevent the Swedish forestry sector from advancing into European standards.

There is a legacy of successful experiences of restoration in Sweden to rely on. The twentieth century has proven the capacity of the Swedish and European forest sector to restore forest land for increased wood biomass and timber production. Now, it is about restoring parts of the forest landscape again, but with a more diversified vision that reflects current and future expectations on sustainable land use in forests. Now, as previously when re-building the Swedish forest sector in the twentieth century, implementing the NRR is not about recreating past conditions, but about creating new conditions and building a landscape that delivers sustainability and a wider range of ecosystem services for the future.
